# Predicting zip code-level vaccine hesitancy in US Metropolitan Areas using machine learning models on public tweets

**DOI:** 10.1371/journal.pdig.0000021

**Published:** 2022-04-07

**Authors:** Sara Melotte, Mayank Kejriwal

**Affiliations:** Information Sciences Institute, University of Southern California, Marina del Rey, California, United States of America; Emory University, UNITED STATES

## Abstract

Although the recent rise and uptake of COVID-19 vaccines in the United States has been encouraging, there continues to be significant vaccine hesitancy in various geographic and demographic clusters of the adult population. Surveys, such as the one conducted by Gallup over the past year, can be useful in determining vaccine hesitancy, but can be expensive to conduct and do not provide real-time data. At the same time, the advent of social media suggests that it may be possible to get vaccine hesitancy signals at an aggregate level, such as at the level of zip codes. Theoretically, machine learning models can be learned using socioeconomic (and other) features from publicly available sources. Experimentally, it remains an open question whether such an endeavor is feasible, and how it would compare to non-adaptive baselines. In this article, we present a proper methodology and experimental study for addressing this question. We use publicly available Twitter data collected over the previous year. Our goal is not to devise novel machine learning algorithms, but to rigorously evaluate and compare established models. Here we show that the best models significantly outperform non-learning baselines. They can also be set up using open-source tools and software.

## Author summary

The rapid development of COVID-19 vaccines has been touted as a miracle of modern medicine and industry-government partnerships. Unfortunately, vaccine hesitancy has been stubbornly high in many countries, including within the United States. Surveys, such as those conducted by organizations such as Gallup, have historically proved to be useful tools with which to poll a representative sample of people on vaccine sentiment, but can be expensive to administer and tend to rely on small sample sizes. As a complementary alternative, we propose to use publicly streaming data from Twitter to quantify vaccine hesitancy at the level of zip-codes in near real-time. Unfortunately, the noise and bias often present in social media begs the practical question of whether a cost-effective approach is feasible. In this article, we propose both a methodology, as well as an experimental study, for addressing these challenges using simple machine learning models. Our goal is not to devise novel algorithms but to rigorously evaluate and compare established models. Using public US-based Twitter data collected in the wake of the pandemic, we find that learning-based models outperform non learning-based models by significant margins. The methods we evaluate can easily be set up using open-source software packages.

## Background

Although more people continue to be vaccinated against COVID-19 in the United States and many other nations with each passing week, significant vaccine hesitancy persists [1, 2]. Vaccine hesitancy in the US has complex drivers, especially among under-served segments of the population [3, 4]. Even prior to COVID-19, vaccine hesitancy against influenza, among other diseases, was non-trivial [5]. In the early days of COVID-19, conspiracy theories about the vaccines had a significant footprint on social media [6, 7]. Such sources of misinformation sometimes go viral on social media, by sowing doubt and mistrust among people who are otherwise undecided about taking the vaccine. Consequently, the potential for real-world public health consequences is very real [7].

Unfortunately, it can be challenging to automatically detect vaccine hesitancy in near-real time. Starting in early 2020, Gallup launched a survey in the United States to study the impacts of COVID-19 from multiple socio-political viewpoints [8]. Such surveys are valuable when released in time for actionable policies and actions to be devised. Due to the dynamic nature of COVID-19 vaccine statistics, it is likely that vaccine hesitancy survey data may be deemed outdated, if released even a few months after the survey is conducted. Reputable survey data can also be expensive to access, and do not provide information in real time. For example, according to a quote available on the Web [9], a 12-month license for the Gallup World Poll Data can cost $30,000.

Given the frenetic pace of digital communication and social media virality [10], more real-time and inexpensive detection of vaccine hesitancy is a well-motivated problem. While the detection needs to be privacy-preserving, the algorithms need to operate at a sufficiently fine-grained spatial granularity, such as at the level of zip codes, to be actionable. Even in a post-COVID era, generalized versions of such systems may help detect and address vaccine hesitancy for a range of diseases, before the hesitancy becomes entrenched in a particular region.

At the same time, recent advances in natural language processing (NLP) and social media analysis have been quite impressive. Using publicly available Application Programming Interfaces (APIs), such as those provided by social media platforms like Twitter [11], high-volume data can be collected inexpensively in real time. We show that, using recent NLP advances, the data can then be processed to yield vaccine hesitancy signals. Although the signals are noisier than carefully collected survey data, their real-time, high-volume and inexpensive nature allow them to serve a complementary role.

Many of the NLP advances we rely on are due to improvements in deep neural networks and language representation learning [12–14]. For example, so-called *word embedding* algorithms, which have been trained on large quantities of text in corpora such as Wikipedia and Google News, learn a real-valued vector representation for each word [13]. We discuss the technical details in more depth in the *Materials and Methods* section. Intuitively, after the word embedding algorithm has finished executing, words that are semantically similar tend to be geometrically closer in the vector space.

Early word embedding algorithms were already capable of analogical reasoning e.g., the vector obtained from the operation $\vec{King}-\vec{Man}+\vec{Woman}$ was found to be closest to the vector $\vec{Queen}$. Impressively, modern variants, proposed in the last 5 years, are now capable of embedding sentences, including tweets, enabling robust machine learning algorithms to be built without manually intensive feature engineering [12, 15].

This article considers the problem of predicting vaccine hesitancy in the United States, using public social media data from Twitter. We focus our study on major metropolitan areas which are known for high tweeting activity, and where users tend to enable the location facility on their phone compared to more rural milieus [16]. We are not looking to predict vaccine hesitancy at an individual level, both due to privacy concerns and also due to problems with accurately evaluating such predictions without polling the individual. Instead, we seek to develop systems that predict vaccine hesitancy at the *zip code-level*.

Specifically, our proposed methods rely on extracting vaccine hesitancy signals from the text in public, geolocated tweets. It does not identify or isolate user data of any kind. An advantage of making predictions at the zip code-level is that predictions can be validated using *independent* survey data, such as the Gallup poll mentioned earlier. As detailed subsequently, by averaging responses of individuals within a given zip code-demarcated region, we are able to obtain a real-valued vaccine hesitancy estimate for that zip code.

To evaluate such estimates, we define and discuss them in the *Materials and Methods* section. Equally important when evaluating such systems is the choice of baselines used for comparisons. In the absence of models that rely on machine learning and social media, a feasible choice might be a system that just predicts a *constant-valued* vaccine hesitancy estimate, sometimes using theoretical models. For instance, the baseline may declare a vaccine hesitancy of 0.5 or 1.0 in a given region. A more sophisticated option is to report the constant representing the average observed in the survey data. Considering such methods in our feasible set of baselines, we show that our proposed machine learning-based models outperform them. The best machine learning model is found to achieve a 10 percent relative improvement over the best constant-valued baseline, which itself relies on privileged information i.e., the mean vaccine hesitancy observed in the survey.

Our models are practical and guided by real-world intuitions. We not only consider the text and hashtags directly observed in geolocated tweets, but also consider the use of NLP software for extracting sentiment signals from the text. Additionally, we explore the use of features from external data sources not grounded in social media, such as the number of hospitals or scientific establishments in a zip code. We experimentally investigate the extent to which the use of these independent sets of features helps in improving the model. In other words, rather than propose a single winning model, we compare a range of models and features-sets to better understand performance differences and tradeoffs.

The rest of this article is structured as follows. We proceed with a comprehensive description of the *Materials and Methods* used in our study. We detail the Twitter dataset and its collection, and subsequent steps, such as data preprocessing and feature extraction. We also discuss the vaccine hesitancy ground-truth that we obtained from independent Gallup survey data. We summarize the evaluation methodology and metrics, and enumerate the models and baselines being evaluated. To enable maximal replicability and minimize cost, we implement our methods using open-source packages and public data. Next, our experimental findings are detailed in *Results*, including statistical significance analyses. A qualitative *Discussion and Error Analysis* section follows. We conclude the work with a summary and brief primer on promising future avenues for research.

## Materials and methods

### Twitter dataset

We sample tweets related to the COVID-19 pandemic from the nine most populous metropolitan areas in the United States [17]. In order of decreasing order of population size, these are: New York, Los Angeles, Chicago, Houston, Phoenix, Philadelphia, San Antonio, San Diego, and Dallas. Our sampled tweets are a subset of the GeoCOV19Tweets dataset [18]. The GeoCOV19Tweets project collected geo-tagged tweets related to the COVID-19 pandemic on a daily basis, using a set of manually determined COVID-specific keywords and hashtags. The project also published a sentiment score for each tweet.

In keeping with Twitter’s terms and conditions, only the tweet ID and sentiment scores were published online. In previous work [19], we *hydrated*, or directly retrieved from Twitter, tweets from the GeoCOV19Tweets dataset dated from March 20, 2020 through December 1, 2020. This period spans a total of 255 days. We skipped the period from October 27, 2020 through October 28, 2020 because sentiment scores were not available in GeoCOV19Tweets during that span.

Next, as discussed in [19], we processed each hydrated *tweet object*, which is a data structure described extensively in Twitter’s developer documentation [20]. Specifically, we extracted a *coordinates* object from this data structure to derive a precise location for the tweet. These coordinates were then used to filter the tweets by metropolitan area, by checking if the coordinates fell within a manually-drawn *bounding box* demarcating each of the metropolitan areas listed earlier.

In this study, we re-hydrate this collection of tweets using the *twarc* library to save the tweet’s full text and tweet ID [21]. After removing any archived tweets, as well as tweets for which the *coordinates* object is no longer available, we retained a total of 45,899 tweets. We also collect each tweet’s zip code of origin by using an Application Programming Interface (API), provided by Geocodio [22]. Founded in 2014, Geocodio provides human-readable location information, such as state, city and country, given a pair of latitude-longitude coordinates as input.

We also eliminate all zip codes with fewer than 10 tweets, resulting in 4,799 tweets and 1,321 zip codes being removed. We then merge the data with the zip code-level attributes described subsequently in *Features from External Sources*. We remove rows with null values, leaving a total of 29,458 tweets, each of which belongs to one of 493 unique zip codes across the nine metropolitan areas listed above. We note that none of the 29,458 tweets is a retweet, allowing for each sample to be treated independently. In Table 1 below, we summarize key statistics of the data, including the number of hashtags in the data both before and after the text preprocessing steps detailed in the next section.

**Table 1:** A summary of key dataset statistics per metropolitan area. Hashtag counts are reported both before, and after, the text preprocessing steps.

Metropolitan Area	Num. Zip Codes	Num. Tweets	Avg. Tweets per Zip Code	Num. Hashtags (before)	Num. Hashtags (after)	Num. Unique Hashtags (after)
New York	152	12,612	82.974	41,232	40,419	10,764
Los Angeles	148	10,532	71.162	37,030	36,507	12,422
Chicago	43	1,544	35.907	3,857	3,792	1,891
Houston	36	1,529	42.472	5,557	5,505	2,526
San Diego	27	1,061	39.296	3,019	2,980	1,769
Philadelphia	24	817	34.042	2,753	2,727	1,349
Dallas	33	664	20.121	2,250	2,231	1,211
Phoenix	20	496	24.80	1,576	1,563	1,003
San Antonio	10	203	20.30	765	756	340
Total	493	29,458	N/A	98,039	96,480	N/A

### Text preprocessing

Using a hydrated tweet’s full text, we tokenize, make lowercase, and remove mentions using *TweetTokenizer* [23] from the Natural Language Toolkit (NLTK) package [24]. NLTK is a leading package in the NLP community that uses Python programs to work with human language data. We also remove URLs, stop words, tokens less than or equal to 1 character in length, and any characters other than letters, including the # symbol and emojis. We use NLTK’s standard set of English stop words [25] e.g., *the*, *a*, and so on. However, we retain the words *not, no, nor, very*, and *most* from this pre-determined set, as these are hypothesized to be relevant for making more accurate vaccine hesitancy predictions.

We then lemmatize all tokens using *WordNetLemmatizer* [26]. A consequence of our text preprocessing steps is that hashtags, such as “covid19”, “covid”, “Covid19”, and “covid-19”, for example, all result in the same token. Furthermore, hashtags consisting of numbers, or single characters only, such as “#2020” or “#K”, are eliminated. In Table 1, the count of hashtags in the tweets, before text preprocessing, is computed by summing the occurrences of # in the full text. After text preprocessing, when the hashtags and text are well-separated and more easily analyzed, we count the number of times a token begins with the # symbol.

Note that we avoid directly using the *hashtags* object embedded within the tweet object for several reasons [27]. First, the object appears to already have applied certain filters e.g., numbers-only strings, such as #2020, are eliminated. Although our text preprocessing steps do so as well, as mentioned above, the *hashtags* object does not accurately represent the number of hashtags in the original tweet. One reason is that it fails to accurately count hashtags in a continuous string. For example, in a tweet from New York containing “…#corona#coronavirus#quarantine#quarantinelife#washyourhands…”, the *hashtags* object was found to be an empty array. Therefore, to carefully control text preprocessing and feature extraction in a replicable and reliable manner, we exclusively use and count hashtags by processing the full text field.

Next, the processed tweets are embedded using the fastText *word embedding* model, which was released by Facebook AI research and contains word vectors trained on English Wikipedia data [28]. The training methodology and specific parameterization is detailed in *Predictive Models and Features*. Herein, we note that a word embedding model is typically a neural network that learns a representation, or *embedding*, of each word in an input text corpus. A classic example of such a word embedding model is word2vec, published almost a decade ago [13]. The embedding is a dense, real-valued vector with a few hundred (or fewer, in some cases) *dimensions*. The number of dimensions is much lower than the *vocabulary* of the corpus, which can be in the tens, if not hundreds, of thousands of unique words. The neural networks underlying these models automatically learn the embeddings by statistically parsing large quantities of text. The idea is that words that are semantically similar will be placed closer together in the vector space.

The fastText model, used in this article, extends and improves the word2vec model by embedding misspelled, or unusually spelled, words, even if it never encountered the specific misspelling during training. This is an obvious benefit when embedding social media text. The model accomplishes this by learning the fine-grained statistical associations between characters in the words, rather than directly learning an embedding for each word. As the name suggests, the model is also optimized to run quickly. It can be used to embed a full sentence or tweet in the vector space, rather than just a word [28]. While an imperfect representation of the tweet’s meaning, we show subsequently that the embedding still contains enough signal that our regression-based models are able to use it to predict vaccine hesitancy within a reasonable margin of error.

### Features from external sources

In the *Twitter Dataset* section, we noted a total of 493 unique zip codes that resulted from including only tweets for which we were able to determine the originating zip code. For each unique zip code, we also collected additional zip code-level information from external, publicly available data sources. These zip code-level attributes, which we add as features in our predictive models, comprise the Zillow Home Value Index (ZHVI) [29], as well as the numbers of establishments in the *educational, healthcare*, and *professional, scientific, or technical* sectors. We incorporate these features as expected, albeit approximate, proxies for measuring affluence and resource availability within a zip code.

As noted earlier, the sentiment features are obtained at the finer-grained granularity of tweets, and were made directly available by the GeoCOV19Tweets project underlying our data [18]. It is important to emphasize that, while each tweet has its own sentiment score, tweets sharing a zip code also share the zip code-level attributes noted above i.e., the zip code-level attributes are repeated for all tweets belonging to the same zip code. Table 2 summarizes each zip code-level, and sentiment. Next, we provide detailed descriptions of these features are provided in the next section.

**Table 2:** External features collected from publicly available data sources. With the exception of the sentiment score, all features are computed as zip code-level attributes, meaning that tweets sharing a zip code will have the same values for these features.

Feature	Description
Sentiment Score	Score between [-1, 1], where positive values signify positive sentiment and 0 signifies a neutral sentiment value. Additionally, the greater the absolute value, the stronger the implied sentiment.
Zillow Home Value Index (ZHVI)	Measure of the typical *home value* (capturing market value, as well as price) in US Dollars within a zip code.
Healthcare and Social Assistance	Number of establishments that provide health care and social assistance to individuals.
Educational Services	Number of establishments, either for-profit or not-for-profit, that provide instruction or training. Such establishments may be privately or publicly owned.
Professional, Scientific, and Technical Services	Number of establishments that provide professional, scientific, and technical services requiring a high level of expertise or training.

#### Sentiment score

We retain the original sentiment scores included in the GeoCOV19Tweets dataset [18] generated using the TextBlob sentiment analysis tool [30]. In this dataset, every tweet is given a continuous value score between [-1, 1], where positive values signify positive sentiment and 0 signifies neutral sentiment. The more positive or negative the value, the stronger the sentiment. Prior to computing these sentiment scores, hashtag symbols (#), mention symbols (@), URLs, extra spaces, and paragraph breaks were eliminated. Punctuation, emojis, and numbers were included.

#### Zillow Home Value Index (ZHVI)

The Zillow Home Value Index (ZHVI) is a measure of the typical home value for a region; in this case, zip code. It captures monthly changes in Zestimates [31], which are Zillow’s estimated home market values that incorporate house characteristics, market data such as listing prices of comparable of homes and their time on the market, as well as off-market data including tax assessment and public records. It also incorporates market appreciation. In this study, we take the average of the smoothened, seasonally adjusted value in the 35th to 65th percentile range (mid-tier) from January through December 2020.

#### Establishments

Data about the number of establishments per zip code is taken from the 2018 Annual Economic Surveys from the US Census (Table ID CB1800ZBP) [32]. We take the *Health care and social assistance, Educational services*, and *Professional, scientific, and technical services* data, which have the following meaning:

1. Healthcare and social assistance (sector 62) comprises establishments providing health care and social assistance for individuals [33] e.g., physician offices, dentists, mental health practitioners, outpatient care centers, ambulance services, etc. [34].
2. Educational services (sector 61) consist of establishments that provide instruction or training in a wide variety of subjects. The sector includes both privately and publicly owned institutions and both for profit and not for profit establishments [35] e.g., elementary and secondary schools, colleges, universities, computer training, professional schools, driving schools, etc. [36].
3. Professional, scientific, and technical services (sector 54) include establishments that specialize in providing professional, scientific, and technical services that require a high level of expertise or training [37] e.g., legal services, notaries, accounting, architectural services, building inspection, engineering services, scientific consulting, research and development, advertising, etc. [38].

All features are normalized using the *StandardScaler* function in Python’s scikit-learn package [39]. Normalization is performed separately within the train and test data splits to prevent any test data leakage into the training phase. The next section provides further details on how the dataset was split into train and test partitions.

### Train /Test split and vaccine hesitancy ground truth

We use stratified splitting, implemented in the *StratifiedShuffleSplit* function in the scikit-learn package [40], to partition our tweets into train (80%) and test (20%) sets. This stratification is applied per zip code, ensuring that both the train and test splits include tweets from all 493 zip codes in approximately equal proportions. For example, 42.8% of both the train and test sets are tweets from the New York City metropolitan area, since 42.8% of the overall tweets in our corpus are from New York, and so on. Due to this stratified construction, both the train and test sets include tweets from all 9 metropolitan areas. Overall, there are 23,566 tweets in the train set and 5,892 tweets in the test set. As the name suggests the train set is used to train the models described in the next section, while the test set is used for evaluations.

In order to evaluate any model, we need to obtain a *ground truth*, defined as the vaccine hesitancy score per zip code that the model is aiming to predict. Therefore, each of the 493 unique zip codes has a single corresponding *vaccine hesitancy* within the *ground truth*. The vaccine hesitancy values range from 0.0 to 1.0 on a continuous scale. Each such per-zip code value represents, on average, how much people are hesitant about the vaccine. It is also an estimate of the percentage of residents, within the zip code, who are vaccine hesitant.

We obtain such a ground truth by leveraging vaccine hesitancy data collected through the COVID-19 Gallup survey [8]. Specifically, Gallup launched a survey on March 13, 2020 that polled people’s responses during the COVID-19 pandemic, using daily random samples of the Gallup Panel. This panel is a probability-based, nationally representative panel of U.S. adults. Vaccine hesitancy was polled by asking a *vaccine hesitancy question*, starting from July 20, 2020 (about four months after the initial survey was launched). The question is worded as follows: *If an FDA-approved vaccine to prevent coronavirus/COVID-19 was available right now at no cost, would you agree to be vaccinated?* A binary response of *Yes* or *No* polls a person’s willingness to be vaccinated. We use the proportion of *No* responses among individuals polled within a specific zip code as our measure of the vaccine hesitancy score for this study. We calculate the proportion of the *No* answers to this question between July 20 and August 30, 2020 at the zip code-level to get a vaccine hesitancy score per zip code.

The mean vaccine hesitancy across all 493 unique zip codes corresponding to our tweets was calculated to be 0.240. The standard deviation is 0.334, showing that there is significant variance across zip codes, even when limited to the largest metropolitan areas in the US. The minimum and maximum values are 0.00 and 1.00, indicating complete vaccine acceptance and hesitancy, respectively.

Note that these ground truth values exist at the zip code-level, and are aggregate measures. A vaccine hesitancy of 0.5 in a zip code intuitively means that, on average, half the people in that zip code are vaccine hesitant. While we cannot say anything about an individual tweeter, for predictive modeling purposes, we *label* a tweet originating from zip code *z* with the ground truth vaccine hesitancy score corresponding to zip code *z*. This implies that, if there are *k* tweets from zip code *z*, then all *k* tweets are assigned the same *pseudo* vaccine hesitancy label. In the next section, we detail this further as an instance of *weakly labeling* the tweets with vaccine hesitancy signals. For completeness, when reporting the findings, we also report metrics at the tweet-level. However, the zip code-level metrics should always be interpreted as the true measure of our system’s performance.

### Evaluation methodology and metrics

All predictive models and baselines used in this study, and described in the next two sections, are evaluated in two different ways: at the tweet-level and at the zip code-level. The tweet-level evaluation is based on a vaccine hesitancy prediction for every tweet in the test set (a total of 5,892 predictions), while the zip code-level evaluation relies on a single vaccine hesitancy prediction per zip code (a total of 493 predictions). Our predictive models, however, only make vaccine hesitancy predictions at the tweet-level. To derive a zip code-level prediction from these tweet-level predictions, we average all the tweet-level predictions within that zip code. Formally, for *k* tweets (in the test set) belonging to zip code *z* with predicted tweet-level vaccine hesitancies $\left[\hat{y_{1}},\ldots ,\hat{y_{k}}\right]$, the predicted vaccine hesitancy for zip code *z* is given by the formula:

$$\begin{array}{c} \hat{y_{z}}=\frac{\sum_{i=1}^{k}\hat{y_{i}}}{k} \end{array}$$ (1)

We use the Root Mean Square Error (RMSE) metric for measuring performance for both tweet-level and zip code-level predictions. Given *m* data points with real-valued ground truth vaccine hesitancy labels [*y*_1_, …, *y*_*m*_], and predicted labels $\left[\hat{y_{1}},\ldots ,\hat{y_{m}}\right]$, the RMSE is given by the formula below:

$$\begin{array}{c} RMSE=\sqrt{\frac{\sum_{i=1}^{m}{\left(y_{i}-\hat{y_{i}}\right)}^{2}}{m}} \end{array}$$ (2)

For the tweet-level evaluation, each of the *m* data points represents a tweet, while for the zip code-level evaluations, each data point represents a zip code. Thus, in the tweet-level RMSE score calculation, the pseudo tweet-level vaccine hesitancy labels, the assignment of which was described in the previous section, are compared with the tweet-level predictions obtained from the model. Similarly, in the zip code-level RMSE calculation, the ground truth vaccine hesitancies, obtained from Gallup, are compared with the zip code-level predictions made by the models. The lower the RMSE score, the lower the predictive error, and the better the model.

We emphasize that, because each zip code is annotated with a real-valued vaccine hesitancy, regression-based predictive modeling applies, rather than classification-based predictive modeling. Hence, we do not consider models that are primarily designed to be used as classifiers, such as Random Forests or Decision Trees. However, future research can potentially consider a different formulation of this problem that enables direct use of classification-based predictors.

Although we train our predictive models at the tweet-level, the tweet-level predictions are auxiliary to obtaining zip code-level vaccine hesitancy predictions. This is because model performance cannot be evaluated at the tweet-level, when our ground truth vaccine hesitancy values are at the zip code-level. In other words, the tweet-level vaccine hesitancy labels should be thought of as *pseudo*, or *weak*, labels. By a *weak* label, we mean that the tweet does not *necessarily* indicate vaccine hesitancy. Namely, the user publishing the tweet is not necessarily vaccine hesitant. Indeed, the tweet may not even be discussing vaccines directly. However, the tweet is published in a zip code for which vaccine hesitancy is known as a real-valued aggregate variable. The intuition is that, for the purposes of modeling, we can assign a tweet *t* published in zip code *z*, the vaccine hesitancy of zip code *z*. The tweet is then said to be weakly labeled with that vaccine hesitancy, since the true vaccine hesitancy of the user publishing the tweet is unknown.

Because weak labeling is, by definition, relatively inaccurate compared to the zip code-level vaccine hesitancy, which is directly derived from survey data, predictive performance at the level of tweets is only reported as an auxiliary result for the sake of completeness. The primary goal of this study, as discussed in *Background*, is to predict zip code-level vaccine hesitancies, using publicly available individual tweets.

In addition to computing a RMSE score on the test set for each predictive model, we also report the average of the 5-fold cross-validated RMSE score at the tweet-level. The methodology is as follows. First, we split the train set into five folds. The first fold contains 4,714 tweets, while the other four folds each contain 4,713 tweets, adding up to a total of 23,566 tweets, which is the entirety of the train set. For the purposes of cross-validation experiments, each fold is used as a *test* set once, while the remaining four folds act as the *train* set.

Because each fold is used as a *test* set only once, there are five training iterations, corresponding to the number of folds. At each iteration, we obtain one RMSE score representing the performance of the model trained on four folds and evaluated on the fifth. Over all iterations, therefore, we have five RMSE scores of which we report the average in *Results* as a measure of model *robustness* i.e., to further verify that the reported tweet-level RMSE values are not the result of luck on the actual test set, containing 5,892 tweets. We also use these scores to do a statistical significance analysis on the best model.

Note that we do not report the average of the 5-fold cross-validated RMSE scores at the zip code-level. The reason is that cross-validation is computed during training, and as mentioned in the previous section, model training is done exclusively at the tweet-level. The sole purpose behind training and cross-validating the predictive models at the tweet-level is to obtain a measure of model robustness, and to enable significance analyses.

### Predictive models and features

As described in *Text Preprocessing*, we use fastText’s word vectors trained on English Wikipedia data to embed tweet text. The resulting vectors are 300-dimensional, and all dimensions are retained throughout the study. We embed the processed full text in three different ways, corresponding to three representation. The first representation includes the *text only* i.e., no hashtags. The second representation includes both *text and hashtags*. Finally, the third representation only considers hashtags if any are available, but reverts back to using the text if no hashtags are present in the tweet. We refer to this last representation as the *hybrid* representation.

For example, ignoring any text transformations discussed in *Text Preprocessing*, the *text only* representation of the tweet “Be back soon my friends #corona #cov19 #notMyVirus #quarantinefitness” would embed only the “Be back soon my friends” part. The *text and hashtags* representation would incorporate the entire tweet, and the *hybrid* representation would embed only “#corona #cov19 #notMyVirus #quarantinefitness”, since this specific tweet contains hashtags. Alternatively, the *hybrid* representation of the tweet “In the hospital not for Corona virus” would embed the tweet’s text because no hashtags are provided. Compared to a representation that uses only hashtags, the hybrid representation is expected to be more robust because it still has the ability to use the text if no hashtags are present.

For each of the three representations described above, we build four predictive models, for a total of 12 models, incorporating all zip code-level features: two support vector regression (SVR) models, a linear regression model, and a stochastic gradient descent (SGD). One of the SVR models uses a radial basis function (RBF) kernel, while the other is based on a linear kernel. All of these models are established regression-based models in the machine learning community. Technical details can be found in any standard text [41].

Using the *SVR with RBF kernel* model, we build three additional predictive models (one per representation) that do not incorporate any zip code-level features. The reason for choosing the *SVR with RBF kernel* model is that, out of all twelve predictive models mentioned above, it was found to perform the best across all representations (subsequently demonstrated in *Results*). Additionally, we evaluate all predictive models, both including and excluding zip code-level features, with and without the sentiment score as feature to understand the impact of sentiment on the RMSE score. Note that the sentiment score is an external, tweet-level feature not computed, or verified, by us, as it is provided directly within the underlying GeoCOV19Tweets dataset.

We set the maximum number of iterations in the *SVR with linear kernel* to 4,000, and specified a random state value of 42 for both the *SVR with linear kernel* and SGD models. Otherwise, we use the default parameters within the *sklearn* library for all predictive models described above. Recall from the previous section that for each of these models, the RMSE score is computed at both the tweet-level and the zip code-level. We also report the mean of the 5-fold cross-validated RMSE scores, applicable only for the tweet-level evaluations.

### Baselines

To evaluate the predictive power of the 15 models described in the previous section, we consider six *constant*-value baselines that predict a single value for each individual tweet (*tweet-level*) and each unique zip code (*zip code-level*). The RMSE scores for the tweet-level baseline predictions are measured with respect to the weakly labeled test set (5,892 tweets), while the errors for the zip code-level baselines are computed with respect to the original zip code-level ground truth (493 zip codes). Our baselines do not rely on sentiment, or on any text or zip code-level features. Table 3 summarizes the models.

**Table 3:** Description of the six constant-value baseline models, along with the corresponding vaccine hesitancy value predicted for all tweets and zip codes, for that baseline.

Baseline System	Description	Constant-Value Vaccine Hesitancy
No vaccine hesitancy	Predicts that none of the zip codes are vaccine hesitant	0.0
Complete vaccine hesitancy	Predicts that all zip codes are completely vaccine hesitant	1.0
Partial vaccine hesitancy	Predicts that all zip codes are partially vaccine hesitant	0.5
Mean pseudo vaccine hesitancy in train set	Predicts that all zip codes have a vaccine hesitancy equal to the average vaccine hesitancy of only the train set tweets (23,566 samples)	0.2349
Mean pseudo vaccine hesitancy for all tweets	Predicts that all zip codes have a vaccine hesitancy equal to the average vaccine hesitancy of all tweets (29,458 samples)	0.2349
Mean vaccine hesitancy in Gallup ground-truth	Predicts that all zip codes have a vaccine hesitancy equal to the average vaccine hesitancy of the ground-truth values (493 samples)	0.2403

Concerning the last three baselines in the tables, both the mean vaccine hesitancy in the train set, and the mean vaccine hesitancy for all tweets, are weighted by the frequencies of the zip codes in our dataset. Additionally, the baseline relying on information from the ground-truth is highly optimistic because it assumes that this information is known. Even the previous two baselines (at the tweet-level) rely on this information since the pseudo-label relies on the zip code-level label, which is obtained from the ground-truth.

In practice, the mean vaccine hesitancy within the ground truth, train set, or the entire dataset, will not be available, since that is what we are aiming at predict at the zip code-level. In subsequent sections, we refer to these baselines as the *optimistic* baselines in contrast with the first three (more realistic) baselines, which assume a manually specified constant value.

## Results

The RMSE metrics tabulated in Table 4 show that all predictive models outperform the best-performing, realistic baseline (*no vaccine hesitancy* with an RMSE of 0.411) at the zip code-level, although not all perform better than the most optimistic baselines (RMSE of 0.334). Specifically, no model has an RMSE score below 0.334, our optimistic baseline value, except for the *SVR with RBF kernel* models (without sentiment). Many of these models (such as the *Text Only Linear Regression* model) can themselves be thought of as baselines, given their prevalence and usage in Twitter-based predictive analytics [42]. We refer to them only as predictive models, however, because they have not been presented in the literature as baselines for the specific purpose of predicting vaccine hesitancy from Twitter data (let alone COVID-19 vaccine hesitancy).

**Table 4:** The Root Mean Square Error (RMSE) scores at both the tweet-level and zip code-level for all models, along with the average of the 5-fold cross-validated RMSE scores for the predictive models. Models specified with (*) do not include any zip code-level features. The other models include all zip code-level features, in addition to text. The RMSE scores for the predictive models, with and without sentiment as a feature, are reported as *without sentiment / with sentiment*. Cross-validation is not applicable for baseline models.

Representation	Model	Tweet-Level RMSE	Mean of 5-fold Cross-Validation RMSE Scores	Zip Code-level RMSE
Text Only	SVR (RBF kernel)	**0.206** / 0.211	**0.209** / 0.214	0.312 / 0.316
	SVR (RBF kernel)*	0.271 / 0.290	0.270 / 0.289	**0.308** / 0.329
	SVR (linear kernel)	0.297 / 0.297	0.295 / 0.295	0.374 / 0.374
	Linear regression	0.280 / 0.280	0.279 / 0.279	0.336 / 0.336
	SGD regressor	0.297 / 0.297	0.299 / 0.299	0.343 / 0.343
Text and Hashtags	SVR (RBF kernel)	0.208 / 0.213	0.210 / 0.215	0.315 / 0.318
	SVR (RBF kernel)*	0.269 / 0.291	0.267 / 0.289	0.314 / 0.335
	SVR (linear kernel)	0.297 / 0.297	0.295 / 0.295	0.374 / 0.373
	Linear regression	0.280 / 0.280	0.278 / 0.278	0.335 / 0.355
	SGD regressor	0.300 / 0.300	0.301 / 0.301	0.343 / 0.343
Hybrid	SVR (RBF kernel)	0.213 / 0.217	0.213 / 0.219	0.319 / 0.320
	SVR (RBF kernel)*	0.287 / 0.287	0.280 / 0.297	0.322 / 0.322
	SVR (linear kernel)	0.303 / 0.303	0.309 / 0.309	0.365 / 0.365
	Linear regression	0.292 / 0.292	0.290 / 0.290	0.334 / 0.334
	SGD regressor	0.307 / 0.307	0.299 / 0.299	0.341 / 0.341
Constant-Value Baselines	No vac. hes.	0.399	N/a	0.411
	Complete vaccine hesitancy	0.830	N/A	0.830
	Partial vaccine hesitancy.	0.418	N/A	0.423
	Mean pseudo vaccine hesitancy in train set	0.322	N/A	0.334
	Mean pseudo vaccine hesitancy for all tweets	0.322	N/A	0.334
	Mean vaccine hesitancy in Gallup ground-truth	0.322	N/A	0.334

For most predictive models, there is no difference in performance when sentiment is included versus when it is excluded. However, *SVR with RBF kernel* shows several noteworthy improvements when sentiment is omitted as a feature. Without sentiment, *SVR with RBF kernel* is the only model that outperforms even the most optimistic baselines across all three representations, regardless of whether zip code-level features are included. The only instance in which the *SVR with RBF kernel* model performs worse than the optimistic baselines, is when the *text and hashtags* representation is used, and when sentiment is included, but all zip code-level features are excluded. Even in this case, the RMSE of 0.335 only represents a 0.1% increase in RMSE, compared with the optimistic baselines.

We observe the lowest zip code-level RMSE score (0.308) for the *SVR with RBF kernel* model (*text only* representation), when no features other than the text are included i.e., no zip code-level features and no sentiment score. This score represents a 7.78% improvement over the optimistic baselines, and a 25.06% improvement over the best-performing, realistic baseline i.e., the *no vaccine hesitancy* baseline. Compared with the *complete vaccine hesitancy* baseline, the model demonstrates a 62.89% improvement, and compared with the *partial vaccine hesitancy* baseline, a 27.19% improvement is observed.

When zip code-level features are added, the *text only* representation achieves an RMSE of 0.312, yielding a 6.59% improvement over the optimistic baselines, and a 24.09% improvement over the *no vaccine hesitancy* baseline. Table 4 shows that adding sentiment to these two models, using the *text only* representation, actually reduces performance at the zip code-level.

Interestingly, adding sentiment as the only external feature to the text embeddings reduces performance greatly for the *text only* and *text and hybrid* representations, when using the *SVR with RBF kernel* model. For the former representation, performance is reduced by 6.38%, while for the latter representation, performance decreases by 6.27%. For the *hybrid* representation, the RMSE score is unaffected.

In addition to the results presented in Table 4, we also built an *SVR with RBF kernel* model that predicts zip code-level vaccine hesitancy, based on zip code-level data only (no text), both with and without sentiment. When predicting vaccine hesitancy based on zip code-level data only, this model achieves an RMSE of 0.332, and when sentiment is added, it achieves an RMSE of 0.333. Both outperform the most realistic *no vaccine hesitancy* baseline model, with 19.22% and 18.98% performance improvements, respectively. They also marginally outperform the optimistic baselines, with 0.60% and 0.30% respective decreases in RMSE scores.

This finding suggests that, even when relying on only the number of establishments in the healthcare, educational, and professional, scientific, or technology sectors, as well as the Zillow Home Value Index (ZHVI), it is possible to predict zip code-level vaccine hesitancy with a marginally lower error, compared with constant-value baselines.

Importantly, the findings show that the addition of tweet text leads to consistently lower error for the zip code-level predictions. The best-performing model shows a 25.06% improvement, compared with only 19.22% when no text is used, over the *no vaccine hesitancy* constant-value baseline. It shows a 62.89% improvement over the *complete vaccine hesitancy* constant-value baseline. Therefore, the use of tweet text, particularly in models implementing the *text only* representation, and without any external features, is a powerful indicator of zip code-level vaccine hesitancy. In other words, the tweet text contains more signal than noise, on average, when predicting vaccine hesitancy.

### Significance analysis

We also conducted a significance analysis of the results in Table 4. Specifically, we took the distribution of the five RMSE scores, achieved on the folds of the cross-validation experiment recorded in Column 4 of Table 4, and computed the sample mean and standard error. Since we are looking to compare the effectiveness of the best model, which is the *Text Only SVR (RBF kernel) without sentiment*, we use the one-sided Student’s t-test. We compute the test statistic and by extension, the p-value, by comparing the sample mean to each model’s mean, both with and without sentiment, as recorded in Column 4 of Table 4.

The corresponding p-values are tabulated in Table 5. The results show that the best model’s performance cannot be explained by chance alone. The results are also fairly robust i.e., the model obtained by adding hashtag-based features is not significantly different from the text-only best model. Similarly, the hybrid version *without* sentiment, as well as the text-only version *with* sentiment, are only moderately different from the best model. Once sentiment is added to the hybrid model, it again becomes significantly worse than the best model.

**Table 5:** One-sided p-values of each model’s Root Mean Square Error (RMSE) score, using the Student’s t-test, against the distribution of per-fold cross-validation means achieved by the best model in Table 4. The best model is the *Text Only SVR (RBF Kernel) without sentiment*, indicated as *Ref* below. The null hypothesis is that the mean of this best reference model is greater than (or in other words, *worse* than) the means shown in Column 4 of Table 4. Models specified with (*) do not include any zip code-level features. The p-values for the predictive models, with and without sentiment as a feature, are reported as *without sentiment / with sentiment*. Since cross-validation is not applicable for the constant-value baseline models, they are excluded in this table.

Representation	Model	p-value
Text Only	SVR (RBF kernel)	Ref / 0.05018
	SVR (RBF kernel)*	7.5123e-06 / 2.55837e-06
	SVR (linear kernel)	1.9186e-06 / 1.9186e-06
	Linear regression	4.3502e-06 / 4.3502e-06
	SGD regressor	1.601e-06 / 1.60098e-06
Text and Hashtags	SVR (RBF kernel)	0.32495 / 0.03205
	SVR (RBF kernel)*	9.175e-06 / 2.55837e-06
	SVR (linear kernel)	1.9186e-06 / 1.9186e-06
	Linear regression	4.6061e-06 / 4.6061e-06
	SGD regressor	1.4668e-06 / 1.4668e-06
Hybrid	SVR (RBF kernel)	0.08037 / 0.006975
	SVR (RBF kernel)*	4.1118e-06 / 1.7508e-06
	SVR (linear kernel)	1.0523e-06 / 1.05228e-06
	Linear regression	2.435e-06 / 2.435e-06
	SGD regressor	1.601e-06 / 1.601e-06

Automatic sentiment analysis, therefore, consistently proves to be a noisy feature for this problem domain. Whether this is due to noise in the sentiment analysis itself, or due to sentiment not being strongly associated with vaccine hesitancy, is an important question for future research to pursue. At minimum, these results indicate that sentiment-based features should be used with caution by computational modelers, when trying to build automatic social media-based vaccine hesitancy detection systems.

## Discussion and error analysis

Although the machine learning-based models presented in the previous sections clearly outperform constant-valued baselines, it is useful to consider the issue from a qualitative lens. Such a lens helps us to understand the kinds of tweets that contain either signal or noise, as well as to hypothesize about sources of prediction error. In this section, we conduct such an analysis by sampling some tweets from the dataset, prior to any text preprocessing. We include only the text and hashtags, i.e. we exclude any mentions, hyperlinks, or locations, for brevity. Tweets sampled from zip codes labeled with a vaccine hesitancy of 1.0 (complete vaccine hesitancy) are enumerated below:

1. “Why is COVID-19 mild for some, deadly for others?” (Chicago)
2. “Corona quarantine day 19. Trapped on the beach. #chickendinner #familytime #corona #coronatine #beachlife” (San Diego)
3. “D.C. residents are encouraged to continue practicing social distancing and take actions to help prevent the spread of COVID-19 [PR] Coronavirus Data Update: March 24” (New York)
4. “New comfortable and colorful masks for coronavirus protection while you are out and about #craftfoundry #covid19masks #covid19masksforsale #coronavirusprotection #coronavirusprotectionmask #cottonmask” (Philadelphia)

We also sampled tweets labeled with a predicted vaccine hesitancy of 0.5 by the best model (partial vaccine hesitancy):

1. “An emotional week for everyone. Everyone keep fighting. Day by day #fighting” (Los Angeles)
2. “Please no close talking [emoji] practice social distancing #corona #codin19 #thistooshallpass #seinfeld #seinfeldmemes #seinfeldquotes” (New York)
3. “good thing the drive-in is open and an easy place for social distancing. #JurassicPark #drivein #movienight #family” (San Antonio)
4. “Covid 19 Testing at First Met! #firstmetchurch #covidtesting #sheilajacksonlee” (Houston)

Finally, we sampled tweets predicted with a vaccine hesitancy of 0.0 (no vaccine hesitancy):

1. “Catch me if you can but don’t get burned. #charmander #pokemon #socialdistancing #corona #onesie” (Chicago)
2. “#GivingGratitude to our #frontlineworkers who are working to keep us safe everyday. #weappreciateyou #covid19 #2020 #amberwavesrealestategroup #kellerwilliamsdmn #weareinthistogether #shelterinplace #stayhome” (Dallas)
3. “This is how you know there’s a problem? When is the last time you saw gas prices this low?! #cov?d19 #corona” (Philadelphia)
4. “I’ve got this pit in my stomach at whats happening in california and all of the USA during this corona virus pandemic especially as homeless with severe epilepsy makes this worse.” (Los Angeles)

These examples illustrate the importance of measuring vaccine hesitancy at an aggregate level, such as zip code, rather than at the individual tweet-level. First, there are obvious ethical and privacy-related concerns with setting up such experiments without individual consent. Second, it is unlikely that extreme cases of vaccine hesitancy or non-hesitancy will manifest on Twitter for the vast majority of the population, although a full study of this hypothesis merits future research. However, at the zip code-level, the predictions are more promising.

As presented earlier in Table 4, we showed that the *text only* representation of the *SVR with RBF kernel* model, excluding sentiment and zip code-level features, achieved an RMSE of 0.308 when predicting vaccine hesitancy at the zip code-level. This model, however, tends to slightly overestimate vaccine hesitancy. In 291 zip codes (out of 493), we find that the predicted vaccine hesitancy is greater than the true vaccine hesitancy. In the remaining 202 zip codes, the predicted vaccine hesitancy is lower than the ground truth value. For 50 zip codes, the predicted vaccine hesitancy is overestimated by 0.20 or more, and when looking at the absolute-value difference between the predicted and true vaccine hesitancies, we find that the gap is 0.20 or more in 179 zip codes.

For instance, in Philadelphia, 83.33% of the zip code-level predictions are over-estimates, the highest among all metropolitan areas. In New York, the metropolitan area with the highest proportion of tweets and the highest average number of tweets per zip code in our study, the predicted vaccine hesitancies for 60.53% of zip codes are over-estimated. In fact, in all metropolitan areas except for Phoenix, San Diego, and Dallas, vaccine hesitancy in more than 50% of the zip codes is over-estimated. In Phoenix, exactly half of all zip code-level vaccine hesitancies are over-estimates. For the remaining metropolitan areas, the percentage, of zip codes having over-estimated vaccine hesitancies falls between 58.11% and 62.79%.

Despite the large proportion of over-estimated vaccine hesitancies presented above, we observe that only 16.67% and 9.21% of the zip codes in Philadelphia and New York, respectively, have over-estimated vaccine hesitancies by a margin greater than 0.20. The metropolitan area with the greatest proportion of zip codes with over-estimated vaccine hesitancies, by 0.20 or more, is San Antonio (30%). San Antonio is also the metropolitan area with the fewest number of tweets. San Diego, on the other hand, has only one zip code (3.70%) where vaccine hesitancy is overestimated by 0.20 points or more.

Despite the large proportion of over-estimates, we observe that Philadelphia has the smallest proportion of zip codes (25%) wherein the absolute-value difference between the predicted and true vaccine hesitancies is higher than 0.20. Thus, using this metric, we note that our *SVR with RBF kernel* model performed best in Philadelphia. On the contrary, the model performs worst in Dallas, with 57.58% of zip codes showing an absolute-value difference, higher than 0.20, between the predicted and true vaccine hesitancies. For all other metropolitan areas, the proportion of zip codes wherein predicted vaccine hesitancy differs by 0.20 or more points from the true vaccine hesitancy falls between 29.73% and 50%.

Overall, the results suggest that the methods presented herein should not be used for highly sensitive predictions, but the low gap between the true hesitancy and predicted hesitancy in many zip codes, especially compared to *constant-valued* baselines, all of which performed worse than the SVR method, suggests that the method can be feasibly used by social scientists and digital health experts as an early warning system.

We close this section with a note on empirically checking the *robustness* of the findings in the previous section. Vaccination has been well underway since the survey data that was used for this study was compiled [43]. One issue that can potentially arise with surveying respondents on their future decisions, is that the responses may have some unknown bias. For instance, due to events that occur in the aftermath of the survey, people may change their opinion.

Given that vaccination data is now available at the zip code-level, at least for large states such as California, one way to do a robustness check of the results is to compare the association between survey-based vaccine hesitancy responses with actual vaccination statistics. We conducted such an analysis by downloading actual full-vaccination rates, as a percentage of the population, from a California state government data portal, the link to which is provided in the *Data Availability* section at the end of the article.

Our specific methodology was to first locate the California zip codes that are common between the Gallup survey data and the actual vaccination data. In total, there were 853 such zip codes. Next, we subtracted 1.0 from the survey-based vaccine hesitancy percentage to obtain a *vaccine proclivity* score. We computed both the Pearson correlation and the Spearman’s rank correlation between vaccine proclivity and the actual vaccination rate. The Pearson correlation was found to be 0.161 (with p-value 2.461e-06) and Spearman’s rank correlation was found to be 0.1664 (with p-value 1.023e-06).

Even a year later, therefore, there is a significant positive correlation between vaccine proclivity and actual vaccination. Although this provides a preliminary robustness check on our ground truth (and by extension, the results that it was used to validate) we leave for future work to conduct a confirmatory assessment of our models, using the actual vaccination rates as the ground truth.

## Conclusion

Although it is declining, significant vaccine hesitancy continues to persist in many geographic and demographic segments of the adult population. Due to the expense of conducting detailed and representative surveys, there is a need for an inexpensive and more real-time predictive model for detecting vaccine hesitancy. In this article, we explored public social media data from Twitter as a potential source of such information. Without identifying individual users, our models use the text and hashtags in tweets to detect vaccine hesitancy at the zip code-level. Using independently collected survey data from Gallup as the ground truth, we conducted an experimental study demonstrating the feasibility of these models.

Specifically, we found that the models performed well compared to constant-valued baselines, despite their being irrelevant and non-vaccine related, or even non-COVID related, tweets, present in the corpus. Using a set of California-based zip codes, we also conducted a robustness check by computing the association between vaccine proclivity, derived from our survey-based ground truth, and the latest full vaccination rates. We found two association measures to be positive and significant, suggesting that the survey data is a reliable ground-truth and a reasonable predictor of vaccination rates in the immediate future.

We end with the caveat that such a system should not be intended as a replacement for comprehensive and representative surveys. Rather, by serving as a supplementary source of information, such a system may help public health officials detect clusters of vaccine hesitancy, and proactively seek to mitigate it with communication and outreach.

Another caveat is that such a system is also expected to be more reliable for urban areas, toward which social media platforms like Twitter tend to be heavily biased in their user-base. Also, our results currently hold for the United States. An important avenue for future research is to replicate and extend these results to other countries, including those where English is not the primary language. If correctly implemented and used, such a system may serve as a valuable and inexpensive asset in a nation’s digital health infrastructure, especially as more people start engaging with social media. We also hope to explore an extension of this work to image- and video-based social media platforms, such as Instagram and TikTok.

## References

[1] Khubchandani J, Sharma S, Price JH, Wiblishauser MJ, Sharma M, Webb FJ. COVID-19 vaccination hesitancy in the United States: a rapid national assessment. J Community Health. 2021 Apr;46(2):270–277. Epub 2021 Jan 3. doi: 10.1007/s10900-020-00958-x 33389421 PMC7778842

[2] United States Office of the Assistant Secretary for Planning and Evaluation. Vaccine hesitancy for COVID-19: PUMA Estimates. 2021 June 16 [Cited 2022 March 2]. Available from: https://aspe.hhs.gov/pdf-report/vaccine-hesitancy-covid-19-puma-estimates.

[3] Fridman A, Gershon R, Gneezy A. COVID-19 and vaccine hesitancy: A longitudinal study. PloS one. 2021 Apr 16;16(4):e0250123. doi: 10.1371/journal.pone.0250123 33861765 PMC8051771

[4] Momplaisir F, Haynes N, Nkwihoreze H, Nelson M, Werner RM, Jemmott J. Understanding drivers of coronavirus disease 2019 vaccine hesitancy among Blacks. Clin Infect Dis. 2021 Nov 16;73(10):1784–1789. doi: 10.1093/cid/ciab102 33560346 PMC7929035

[5] Kempe A, Saville AW, Albertin C, Zimet G, Breck A, Helmkamp L, Vangala S, Dickinson LM, Rand C, Humiston S, Szilagyi PG Parental hesitancy about routine childhood and influenza vaccinations: a national survey. Pediatrics. 2020 Jul;146(1):e20193852. Epub 2020 Jun 15. doi: 10.1542/peds.2019-3852 32540985 PMC7329256

[6] Brennen JS, Simon F, Howard PN, Nielsen RK. Types, sources, and claims of COVID-19 misinformation. Doctoral Dissertation, University of Oxford. 2020. Available from: https://reutersinstitute.politics.ox.ac.uk/sites/default/files/2020-04/Brennen%20-%20COVID%2019%20Misinformation%20FINAL%20(3).pdf.

[7] Enders AM, Uscinski JE, Klofstad C, Stoler J. The different forms of COVID-19 misinformation and their consequences. The Harvard Kennedy School Misinformation Review. 2020. Available from: https://dash.harvard.edu/bitstream/handle/1/37366466/enders_covid_19_misinformation_consequences_20201116.pdf?sequence=1.

[8] Brenan M. Roundup of Gallup COVID-19 coverage. 2022 Jan 11 [Cited 2022 March 2]. Available from: https://news.gallup.com/opinion/gallup/308126/roundup-gallup-covid-coverage.aspx.

[9] The Gallup Organization. Gallup World Poll data license. 2022 [Cited 2022 March 2]. Available from: https://aws.amazon.com/marketplace/pp/prodview-uapupqnfizgci#offers.

[10] Varis P, Blommaert J. Conviviality and collectives on social media: Virality, memes, and new social structures. Multilingual Margins: A journal of multilingualism from the periphery. 2015;2(1):31–31.

[11] Makice K. Twitter API: Up and running: Learn how to build applications with the Twitter API. O’Reilly Media, Inc.; 2009 Mar 17.

[12] Devlin J, Chang MW, Lee K, Toutanova K. Bert: Pre-training of deep bidirectional transformers for language understanding. arXiv:181004805 [Preprint]. 2019 May 24 [Cited 2022 March 2]. Available from: https://arxiv.org/pdf/1810.04805.pdf&usg=ALkJrhhzxlCL6yTht2BRmH9atgvKFxHsxQ.

[13] Mikolov T, Grave E, Bojanowski P, Puhrsch C, Joulin A. Advances in pre-training distributed word representations. arXiv:171209405 [Preprint]. 2017 Dec 26 [Cited 2022 March 2]. Available from: https://arxiv.org/pdf/1712.09405.pdf.

[14] Joulin A, Grave E, Bojanowski P, Mikolov T. Bag of tricks for efficient text classification. arXiv:160701759 [Preprint]. 2016 Jul 6 [Cited 2022 March 2]. Available from: https://arxiv.org/pdf/1607.01759.pdf%E3%80%82%E8%AE%BA%E6%96%87%E9%9D%9E%E5%B8%B8%E7%9F%AD%EF%BC%8C%E5%8A%A0%E4%B8%8AReferences%E4%B8%8D%E8%BF%87%E4%BA%94%E9%A1%B5%EF%BC%8CModel.

[15] Floridi L, Chiriatti M. GPT-3: Its nature, scope, limits, and consequences. Minds and Machines. 2020 Dec;30(4):681–694. doi: 10.1007/s11023-020-09548-1

[16] Malik M, Lamba H, Nakos C, Pfeffer J. Population bias in geotagged tweets. In proceedings of the international AAAI conference on web and social media 2015 (Vol. 9, No. 4, pp. 18–27). Available from: https://ojs.aaai.org/index.php/ICWSM/article/download/14688/14537.

[17] Ballotpedia. Largest Cities in the United States by Population. 2020 [Cited 2022 March 2]. Available from: https://ballotpedia.org/Largest_cities_in_the_United_States_by_population.

[18] Lamsal R. Coronavirus (covid-19) tweets dataset; 2020 [cited 2022 March 2]. Database: IEEE Dataport [Internet]. Available from: 10.21227/781w-ef42.

[19] Melotte S, Kejriwal M. A Geo-Tagged COVID-19 Twitter Dataset for 10 North American Metropolitan Areas over a 255-Day Period. Data. 2021 Jun 14;6(6):64. doi: 10.3390/data6060064

[20] Twitter. Twitter Developer Platform: Docs. [Cited 2022 March 2]. Available from: https://developer.twitter.com/en/docs.

[21] Twarc. Collect Twitter Data with Twarc! [Cited 2022 March 2]. Available from: https://scholarslab.github.io/learn-twarc/.

[22] Geocodio. Geocodio Features. [Cited 2022 March 2]. Available from: https://www.geocod.io/features/api/.

[23] Natural Language Tool Kit. Documentation: nltk.tokenize package. 2022 Feb 9 [Cited 2022 March 2]. Available from: https://www.nltk.org/api/nltk.tokenize.html.

[24] Natural Language Tool Kit. Documentation: Natural Language Toolkit. 2022 Feb 9 [Cited 2022 March 2]. Available from: https://www.nltk.org/index.html.

[25] Natural Language Tool Kit. NLTK Corpora. 2022 Feb 9 [Cited 2022 March 2]. Available from: http://www.nltk.org/nltk_data/.

[26] Natural Language Tool Kit. Documentation: nltk.stem package. 2022 Feb 9 [Cited 2022 March 2]. Available from: http://www.nltk.org/api/nltk.stem.html.

[27] Twitter. Data dictionary: Standard v1.1. [Cited 2022 March 2]. Available from: https://developer.twitter.com/en/docs/twitter-api/v1/data-dictionary/object-model.

[28] Facebook Research. FastText pre-trained vectors; 2019 [cited 2022 March 2]. Database: GitHub Iinternet]. Available from: https://github.com/facebookresearch/fastText/blob/master/docs/pretrained-vectors.md.

[29] Zillow. Zillow Home Value Index (ZHVI) user guide. [Cited 2022 March 2]. Available from: https://www.zillow.com/research/zhvi-user-guide/.

[30] Loria S. textblob Documentation. Release 015. 2018 Dec;2:269. Available from: https://media.readthedocs.org/pdf/textblob/latest/textblob.pdf.

[31] Zillow. Zillow Home Value Index (ZHVI) methodology. [Cited 2022 March 2]. Available from: https://www.zillow.com/research/zhvi-methodology-2019-highlights-26221/.

[32] United States Census Bureau. All Sectors: ZIP Code Business Patterns by Employment Size Class for 5-digit zipcode level; 2018 [cited 2022 March 2]. Available from: https://data.census.gov/cedsci/table?q=CB1800ZBP&tid=ZBP2018.CB1800ZBP.

[33] United States Bureau of Labor Statistics. Establishments providing health care and social assistance for individuals. [Cited 2022 March 2]. Available from: https://www.bls.gov/iag/tgs/iag62.htm.

[34] United States Census Bureau. NAICS: Healthcare. [Cited 2022 March 2]. Available from: https://www.census.gov/naics/?input=62&chart=2017.

[35] United States Bureau of Labor Statistics. Establishments that provide instruction or training in a wide variety of subjects. [Cited 2022 March 2]. Available from: https://www.bls.gov/iag/tgs/iag61.htm.

[36] United States Census Bureau. NAICS: Education. [Cited 2022 March 2]. Available from: https://www.census.gov/naics/?input=61&chart=2017.

[37] United States Bureau of Labor Statistics. Establishments that specialize in providing professional, scientific, and technical services that require a high level of expertise or training. [Cited 2022 March 2]. Available from: https://www.bls.gov/iag/tgs/iag54.htm.

[38] United States Census Bureau. NAICS: Professional, Scientific and Technical Services. [Cited 2022 March 2]. Available from: https://www.census.gov/naics/?input=54&chart=2017.

[39] Scikit learn. Documentation: sklearn.preprocessing.StandardScaler. [Cited 2022 March 2]. Available from: https://scikit-learn.org/stable/modules/generated/sklearn.preprocessing.StandardScaler.html.

[40] Scikit learn. Documentation: sklearn.model_selection.StratifiedShuffleSplit. [Cited 2022 March 2]. Available from: https://scikit-learn.org/stable/modules/generated/sklearn.model_selection.StratifiedShuffleSplit.html.

[41] Bishop CM, Nasrabadi NM. Pattern recognition and machine learning. New York: springer; 2006 Aug 17.

[42] Kursuncu U, Gaur M, Lokala U, Thirunarayan K, Sheth A, Arpinar I. B. Predictive analysis on Twitter: Techniques and applications. In Emerging research challenges and opportunities in computational social network analysis and mining 2019 (pp. 67–104). Springer Cham.

[43] Shah ASV, Gribben C, Bishop J, Hanlon P, Caldwell D, Wood R, Reid M, McMenamin J, Goldberg D, Stockton D, Hutchinson S, Robertson C, McKeigue PM, Colhoun HM, McAllister DA Effect of vaccination on transmission of SARS-CoV-2. N Engl J Med. 2021 Oct 28;385(18):1718–1720. Epub 2021 Sep 8. doi: 10.1056/NEJMc2106757 34496200 PMC8451182

